# Risk Analysis Index Outperforms the Modified Frailty Index in Predicting Outcomes in Thyroidectomy and Parathyroidectomy

**DOI:** 10.1002/ohn.70125

**Published:** 2026-01-19

**Authors:** Akshay Warrier, Sruthi Ranganathan, Deondra Montgomery, Jonathan Tawil, Ari Istanboulli, Christian Bowers, Richard K. Gurgel, Hilary McCrary

**Affiliations:** ^1^ Department of Otolaryngology Rutgers New Jersey Medical School Newark New Jersey USA; ^2^ Bowers Neurosurgical Frailty and Outcomes Data Science Lab Sandy Utah USA; ^3^ Department of Surgery, School of Clinical Medicine University of Cambridge Cambridge UK; ^4^ Department of Otolaryngology, College of Human Medicine Michigan State University East Lansing Michigan USA; ^5^ Department of Otolaryngology American University of the Caribbean School of Medicine Cupecoy Sint Maarten; ^6^ Department of Otolaryngology University of Utah Salt Lake City Utah USA

**Keywords:** frailty, mFI‐5, parathyroidectomy, RAI, thyroidectomy

## Abstract

**Objective:**

In an aging population, patients undergoing thyroidectomy and parathyroidectomy are at an increased risk of adverse outcomes; thus, measuring patient frailty is a key metric to assess risk. This study innovatively compares the utility of the Risk Analysis Index (RAI) with the 5‐factor Modified Frailty Index (mFI‐5) in predicting adverse postoperative outcomes.

**Study Design:**

Retrospective cohort.

**Setting:**

US hospitals.

**Methods:**

Patients undergoing thyroidectomy or parathyroidectomy procedures were selected from the 2005 to 2020 NSQIP data set. RAI and mFI‐5 frailty scores were calculated and stratified: non‐frail (RAI: <21/mFI‐5: <1), pre‐frail (RAI: 21‐30/mFI‐5: 1), frail (RAI: 31‐40/mFI‐5: 2), and severely frail (RAI: 40+/mFI‐5: 3‐5) categories. Univariate and multivariate analyses were conducted, followed by receiver operating characteristic (ROC) curves, to evaluate the comparative discriminative thresholds of the indices.

**Results:**

A cohort of 30,362 patients was identified with a median age of 56 years. Multivariate odds ratios showed that both indices were significant independent predictors of mortality (RAI: 15.508, *P* < .001; mFI‐5: 10.713, *P* < .001), extended length of stay (eLOS) (RAI: 9.480, *P* < .001; mFI‐5: 7.952, *P* < .001), non‐home discharge (RAI: 15.897, *P* < .001; mFI‐5: 9.346, *P* < .001), and Clavien‐Dindo (CD) II complications (RAI: 7.130, *P* < .001; mFI‐5: 3.760, *P* < .001). ROC analysis demonstrated significantly superior discrimination by the RAI for mortality (0.769 vs 0.650, *P* = .022), eLOS (0.712 vs 0.596, *P* < .001), non‐home discharge (0.763 vs 0.639, *P* < .001), CD II (0.739 vs 0.566, *P* < .001), CD IIIb (0.644 vs 0.587, *P* = .002), CD IV (0.707 vs 0.622, *P* < .001), and organ/space infection (0.719 vs 0.519, *P* < .001).

**Conclusion:**

Both the RAI and mFI‐5 frailty indices are comparable, significant predictors of adverse events in thyroidectomy/parathyroidectomy. The RAI demonstrated superior discrimination for predicting postoperative morbidity across most outcomes, indicating it may be a superior clinical tool for identifying high‐risk patients. The RAI may better inform perioperative decision‐making, patient counseling, and resource allocation.

*Level of Evidence*: 3.

Frailty, defined as a decline in baseline physiological reserve, is a pivotal determinant of surgical risk, impacting outcomes across various procedures, including those in the head and neck. This syndrome, which comprises decreased strength, impaired cognition, and comorbidities, predisposes patients to postoperative complications due to increased vulnerability to stressors. While age has traditionally been associated with surgical risk, frailty indices, such as the Modified Frailty Index (mFI) and Risk Analysis Index (RAI), provide a more nuanced view of patient resilience and are more predictive of outcomes than age alone.^1^, ^2^

In thyroid and parathyroid surgeries, where patients may present with diverse health profiles and age ranges, the utility of frailty‐based risk stratification is especially relevant. For instance, given the excellent prognosis of well‐differentiated thyroid cancer and its minimal impact on mortality, frailty metrics can guide surgical decision‐making by identifying patients who may face greater harm from aggressive interventions than benefit, as their frailty—not cancer—is more likely to determine outcomes.^3^, ^4^ Even in lower‐risk surgeries, frailty remains important because minor perioperative stressors can overwhelm patients with limited physiological reserve. Unlike age, frailty predicts complications independent of procedural complexity and serves as a quantitative marker of risk that supports shared decision‐making and guides personalized perioperative optimization.

The RAI, which integrates variables covering physical functionality, cognitive status, and comorbidities, allows clinicians to capture a more comprehensive picture of patient risk than traditional comorbidity scores. Despite extensive research on RAI's role in neurosurgical and orthopedic surgery contexts, its application in otolaryngology, and specifically thyroid and parathyroid procedures, remains underexplored.^5^ Thus, assessing the RAI in this low‐risk population is an important litmus test for frailty tools. Establishing whether the RAI outperforms the mFI‐5 in this setting is clinically meaningful, because risk prediction in low‐acuity surgery increasingly guides ambulatory triage, perioperative optimization, and shared decision‐making.

With an aging population, effective preoperative assessments for thyroid and parathyroid surgeries are critical to improving outcomes and aligning perioperative care with patient needs. This study aims to evaluate the RAI's predictive accuracy for postoperative complications and mortality in thyroid and parathyroid surgeries, laying a foundation for broader application across surgeries within the field of otolaryngology and other low‐risk cohorts.

## Methods

### Data Source

Patient‐level data were obtained from the American College of Surgeons (ACS) National Surgical Quality Improvement Program (NSQIP) Participant Use Data Files for the years 2015 through 2020. The ACS NSQIP is a prospectively maintained, risk‐adjusted database that captures perioperative information across more than 700 hospitals in the United States. More than 200 preoperative, intraoperative, and 30‐day postoperative variables are recorded for surgical patients, with abstraction performed by trained clinical reviewers to ensure reliability and uniformity.^2^, ^6^ The study was conducted in a fashion similar to Bowers et al (2023) and used Health Insurance Portability and Accountability Act–compliant files, qualified as exempt by the Rutgers New Jersey Medical School institutional review board.^2^

### Patient Selection

Eligible patients were identified using the Current Procedural Terminology (CPT) codes listed in Supplemental Table S1, available online. Inclusion criteria were age ≥ 18 years and a qualifying procedure performed between 2005 and 2020. Patients were excluded if they were older than 90 years (n = 42, 0. 0.14%), as exact age above 90 was not coded in the database; had missing functional status (n = 193, 0.6%); or lacked other essential clinical data. After exclusions, 30,362 patients remained in the analytic cohort. Separate thyroid‐only and parathyroid‐only subgroup analyses were considered; however, >93% of cases carried combined thyroid/parathyroid CPT coding, precluding stable, independent modeling. Patients were stratified into four frailty categories—non‐frail, pre‐frail, frail, and severely frail—based on both the RAI and the 5‐factor Modified Frailty Index (mFI‐5) (Table 1).

**Table 1:** Components of Frailty Indices in Thyroid/Parathyroid Surgery and Associated Scoring

Modified frailty index 5 (mFI‐5)	Risk analysis index (RAI)
1. Diabetes mellitus (+1)	1. Age
2. Hypertension (+1)	2. Sex (female = 0, male = +5)
3. Dependent functional status (+1)	3. Cancer diagnosis excluding melanoma (range 13‐20)
4. Chronic obstructive pulmonary disease or pneumonia (+1)	4. Unintentional weight loss of 4.5 kg over 3 mo (+5)
5. Congestive heart failure (+1)	5. Renal failure or dialysis (+6)
	6. Congestive heart failure (+4)
	7. Poor appetite (+4)
	8. Shortness of breath at rest or minimal activity (+8)
	9. Residence other than independent living (+8)
	10. Cognitive deterioration
	11. Activities of daily living (ADLs) defined as functional status:
	a. Mobility (0‐4)
	b. Eating (0‐4)
	c. Toilet use (0‐4)
	d. Hygiene (0‐4)

### Five‐Factor Modified Frailty Index

The mFI was originally introduced as an 11‐item score (mFI‐11). In 2014, the ACS NSQIP discontinued the collection of six of these variables, leading to the development of the 5‐item version (mFI‐5). The mFI‐5 incorporates functional status, diabetes mellitus, chronic obstructive pulmonary disease, hypertension, and congestive heart failure (Supplemental Table S1, available online).^2^ Each factor contributes one point, resulting in scores from 0 to 5. Patients with scores of 0 are classified as non‐frail, 1 as pre‐frail, 2 as frail, and ≥3 as severely frail. Both the mFI‐11 and mFI‐5 have been shown to predict adverse outcomes following diverse surgical procedures.^7^, ^8^, ^9^, ^10^

### Risk Analysis Index

The RAI was developed to enhance preoperative frailty assessment and is available in two forms: the prospective RAI (RAI‐C) and the retrospective administrative RAI (RAI‐A).^6^ The instrument evaluates factors (age, sex), comorbid conditions (eg, heart failure, renal disease, dyspnea, and cancer), nutritional status (eg, recent weight loss), and physical and cognitive function, including activities of daily living (ADLs).^1^, ^2^, ^6^ The RAI‐C is more sensitive but less specific due to its reliance on patient self‐report, while the RAI‐A uses standardized administrative definitions derived from NSQIP and is considered more stringent. For the current analysis, the RAI‐A was applied, with scoring thresholds consistent with prior literature: non‐frail (≤10), pre‐frail (11–20), frail (21–30), and severely frail (≥31) (Supplemental Table S2, available online).^2^, ^5^, ^6^, ^7^, ^8^, ^9^, ^10^, ^11^ Preoperative cognitive decline, as well as certain NSQIP fields no longer collected (eg, neoadjuvant radiotherapy/chemotherapy), were excluded, as their absence does not materially affect model performance.^2^, ^6^

### Outcomes and Complications

Primary outcomes included 30‐day mortality, Clavien‐Dindo (CD I, CD II, CD IIIb, and CD IV) complications, 30‐day unplanned readmission, 30‐day unplanned reoperation, extended length of stay (eLOS), and non‐home discharge (NHD). CD complications were categorized according to the published classification (Supplemental Table S2, available online). eLOS was defined as hospitalization beyond the 75th percentile for the cohort, corresponding to >4 days. Secondary outcomes included superficial surgical site infection, deep incisional or organ/space infection, and wound dehiscence.^12^

### Statistical Analysis

Analyses were performed using IBM SPSS Statistics version 28.0 (IBM Corp.) and R version 3.5.3 (The R Foundation for Statistical Computing). Continuous variables with non‐normal distributions are summarized as medians with interquartile ranges (IQRs). Binary logistic regression models were used for univariate and multivariable analysis of postoperative outcomes, with age, mFI‐5, and RAI included as predictors, and the non‐frail cohort as reference. Results are presented as odds ratios (ORs) with 95% confidence intervals (CIs).^2^ To account for potential multicollinearity due to overlap between frailty components, variance inflation factors (VIFs) were assessed for all predictors. Variables with a VIF > 5 were excluded from multivariable models. Discrimination of frailty models was evaluated using receiver operating characteristic (ROC) curve analysis, and differences in *C*‐statistics were compared with the DeLong test. Statistical significance was defined as *P* < .05.

## Results

### Study Population Characteristics

There were 30,362 patients included in this study, with a median age of 56 (IQR = 22), where 76% of the population was female, and the majority were white (58.8%, n = 17,864). Other stratified baseline demographic factors, postoperative outcomes, and univariate analysis are summarized in Table 2. The three most common comorbidities identified were hypertension (48.0%, n = 14,563), dyspnea (7.9%, n = 2397), and requiring dialysis (5.6%, n = 1697). Most patients in the included cohort were functionally independent (85.1%, n = 25,839) and had elective surgery (78.7%, n = 23,909). The RAI score distribution was as follows: non‐frail, 38.58% (n = 20,332); pre‐frail, 17.84% (n = 8782); frail, 2.28% (n = 691); and severely frail, 0.14% (n = 42). The mFI‐5 score distribution was as follows: non‐frail, 41.67% (n = 12,648); pre‐frail, 38.87% (n = 11,798); frail, 17.78% (n = 5396); and severely frail, 1.71% (n = 520). The most common poor postoperative outcomes noted were eLOS (2.0%, n = 597), CD IIIb (1.6%, n = 499), and any wound complication (1.2%, n = 374).

**Table 2:** Demographic, Clinical, and Perioperative Characteristics of Adult Thyroid and Parathyroid Surgery Patients Characterized by Frailty Screening Tool (Risk Analysis Index [RAI] and 5‐Factor Modified Frailty Index [mFI‐5], n = 30,362)

		RAI categories				mFI‐5 categories			
Category	Totals n = 30,362	Non‐frail n = 20,332	Pre‐frail n = 8782	Frail n = 1175	Severely frail n = 73	Non‐frail n = 12,648	Pre‐frail n = 11,798	Frail n = 5396	Severely frail n = 520
*Demographics*									
Age, median (IQR), y	56 (22)	50 (19)	69 (12)	74 (14)	75 (16)	49 (22)	59 (19)	63 (16)	64 (16)
Male, n (%)	7315	3414 (11.24%)	3288 (10.83%)	566 (1.86%)	47 (0.15%)	2547 (20.14%)	3118 (26.43%)	1480 (27.43%)	170.0 (32.69%)
Race, n (%)									
White, n (%)	17,864	11,715 (38.58%)	5416 (17.84%)	691 (2.28%)	42 (0.14%)	8080 (63.88%)	6883 (58.34%)	2655 (49.20%)	246.0 (47.31%)
Black, n (%)	4253	3091 (10.18%)	1046 (3.45%)	107 (0.35%)	9 (0.03%)	1102 (8.71%)	1996 (16.92%)	1042 (19.31%)	113.0 (21.73%)
Asian, n (%)	1019	820 (2.70%)	173 (0.57%)	23 (0.08%)	3 (0.01%)	547 (4.32%)	313 (2.65%)	156 (2.89%)	3.0 (0.58%)
Hispanic, n (%)	2063	1693 (5.58%)	306 (1.01%)	59 (0.19%)	5 (0.02%)	1071 (8.47%)	654 (5.54%)	319 (5.91%)	19.0 (3.65%)
Other,^a^ n (%)	5163	3013 (9.92%)	1841 (6.06%)	295 (0.97%)	14 (0.05%)	1848 (14.61%)	1952 (16.55%)	1224 (22.68%)	139.0 (26.73%)
*Preoperative clinical status*									
Hypertension, n (%)	14,563	7769 (25.59%)	5961 (19.63%)	777 (2.56%)	56 (0.18%)	8733 (69.05%)	5311 (45.02%)	519 (9.62%)	519 (99.8%)
CHF, n (%)	158	31 (0.10%)	81 (0.27%)	38 (0.13%)	8 (0.03%)	12 (0.09%)	71 (0.60%)	75 (1.39%)	75 (14.4%)
COPD, n (%)	872	299 (0.98%)	453 (1.49%)	108 (0.36%)	12 (0.04%)	215 (1.70%)	396 (3.36%)	261 (4.84%)	261 (50.2%)
Dyspnea, n (%)	2397	777 (2.56%)	1300 (4.28%)	285 (0.94%)	35 (0.12%)	506 (4.00%)	933 (7.91%)	763 (14.14%)	195.0 (37.50%)
Renal failure, n (%)	120	72 (0.24%)	43 (0.14%)	5 (0.02%)	0 (0.00%)	16 (0.13%)	59 (0.50%)	31 (0.57%)	14.0 (2.69%)
Dialysis, n (%)	1697	1130 (3.72%)	495 (1.63%)	71 (0.23%)	1 (0.00%)	189 (1.49%)	884 (7.49%)	509 (9.43%)	115.0 (22.12%)
Disseminated cancer, n (%)	475	2 (0.01%)	33 (0.11%)	386 (1.27%)	54 (0.18%)	222 (1.76%)	179 (1.52%)	66 (1.22%)	8.0 (1.54%)
Weight loss, n (%)	367	47 (0.15%)	163 (0.54%)	126 (0.41%)	31 (0.10%)	110 (0.87%)	150 (1.27%)	90 (1.67%)	17.0 (3.27%)
Non‐home origin, n (%)	249	98 (0.32%)	86 (0.28%)	53 (0.17%)	12 (0.04%)	63 (0.50%)	83 (0.70%)	65 (1.20%)	38.0 (7.31%)
Functional status, n (%)									
Independent, n (%)	26,031	18,905 (62.27%)	6335 (20.86%)	568 (1.87%)	31 (0.10%)	12,577 (99.44%)	9638 (81.69%)	3428 (63.53%)	38 (7.30%)
Partial dependence, n (%)	4283	1299 (4.28%)	2386 (7.86%)	565 (1.86%)	33 (0.11%)	2062 (16.30%)	1916 (16.24%)	305 (5.65%)	196 (37.69%)
Total dependence, n (%)	47	5 (0.02%)	8 (0.03%)	25 (0.08%)	9 (0.03%)	7 (0.06%)	27 (0.23%)	13 (0.24%)	305 (58.7%)
Elective surgery status, n (%)	23,909	17,500 (57.64%)	5820 (19.17%)	557 (1.83%)	32 (0.11%)	11,674 (92.30%)	8834 (74.88%)	3169 (58.73%)	232.0 (44.62%)
*Surgical category*									
Thyroid surgery only, n (%)	630	220 (1.08%)	312 (3.54%)	92 (7.82%)	6 (8.22%)	119 (0.94%)	267 (2.26%)	218 (4.03%)	26 (4.97%)
Parathyroid surgery only, n (%)	1078	444 (2.18%)	531 (6.03%)	98 (8.33%)	5 (6.85%)	108 (0.85%)	545 (4.61%)	377 (6.97%)	48 (9.18%)
Thyroid and parathyroid, n (%)	28,366	19,578 (96.17%)	7793 (88.51%)	939 (79.78%)	56 (76.71%)	12,315 (97.28%)	10,866 (91.89%)	4747 (87.81%)	438 (83.75%)
*Postoperative outcomes*									
Any wound complication, n (%)	374	180 (0.59%)	142 (0.47%)	40 (0.13%)	12 (0.04%)	136 (1.08%)	152 (1.29%)	72 (1.33%)	14.0 (2.69%)
SSI, n (%)	196	106 (0.35%)	69 (0.23%)	15 (0.05%)	6 (0.02%)	73 (0.58%)	75 (0.64%)	40 (0.74%)	8.0 (1.54%)
DSSI, n (%)	66	34 (0.11%)	21 (0.07%)	9 (0.03%)	2 (0.01%)	23 (0.18%)	30 (0.25%)	10 (0.19%)	3.0 (0.58%)
OSSI, n (%)	69	21 (0.07%)	34 (0.11%)	11 (0.04%)	3 (0.01%)	27 (0.21%)	27 (0.23%)	12 (0.22%)	3.0 (0.58%)
Dehisc., n (%)	83	32 (0.11%)	35 (0.12%)	13 (0.04%)	3 (0.01%)	20 (0.16%)	42 (0.36%)	15 (0.28%)	6.0 (1.15%)
Clavien‐Dindo II, n (%)	327	97 (0.32%)	166 (0.55%)	51 (0.17%)	13 (0.04%)	102 (0.81%)	138 (1.17%)	72 (1.33%)	15.0 (2.88%)
Clavien‐Dindo IIIb, n (%)	499	261 (0.86%)	181 (0.60%)	48 (0.16%)	9 (0.03%)	166 (1.31%)	223 (1.89%)	92 (1.70%)	18.0 (3.46%)
Clavien‐Dindo IV, n (%)	310	117 (0.39%)	143 (0.47%)	44 (0.14%)	6 (0.02%)	72 (0.57%)	133 (1.13%)	81 (1.50%)	24.0 (4.62%)
Mortality, n (%)	53	16 (0.05%)	22 (0.07%)	13 (0.04%)	2 (0.01%)	12 (0.09%)	20 (0.17%)	11 (0.20%)	10.0 (1.92%)
eLOS, n (%)	597	217 (0.71%)	263 (0.87%)	98 (0.32%)	19 (0.06%)	170 (1.34%)	237 (2.01%)	142 (2.63%)	48.0 (9.23%)
Non‐home discharge, n (%)	340	109 (0.36%)	151 (0.50%)	71 (0.23%)	9 (0.03%)	97 (0.77%)	131 (1.11%)	83 (1.54%)	29.0 (5.58%)

Abbreviations: CHF, congestive heart failure; COPD, chronic obstructive pulmonary disease; Dehisc., wound dehiscence; DSSI, deep incisional site infection; eLOS, extended length of stay; IQR, interquartile range; OSSI, organ/space surgical site infection; SSI, superficial incisional site infection.

^a^ Unknown/Native Hawaiian or Other Pacific Islander/American Indian or Alaska Native.

### Multivariate Regression Analysis

**Figure 1:**
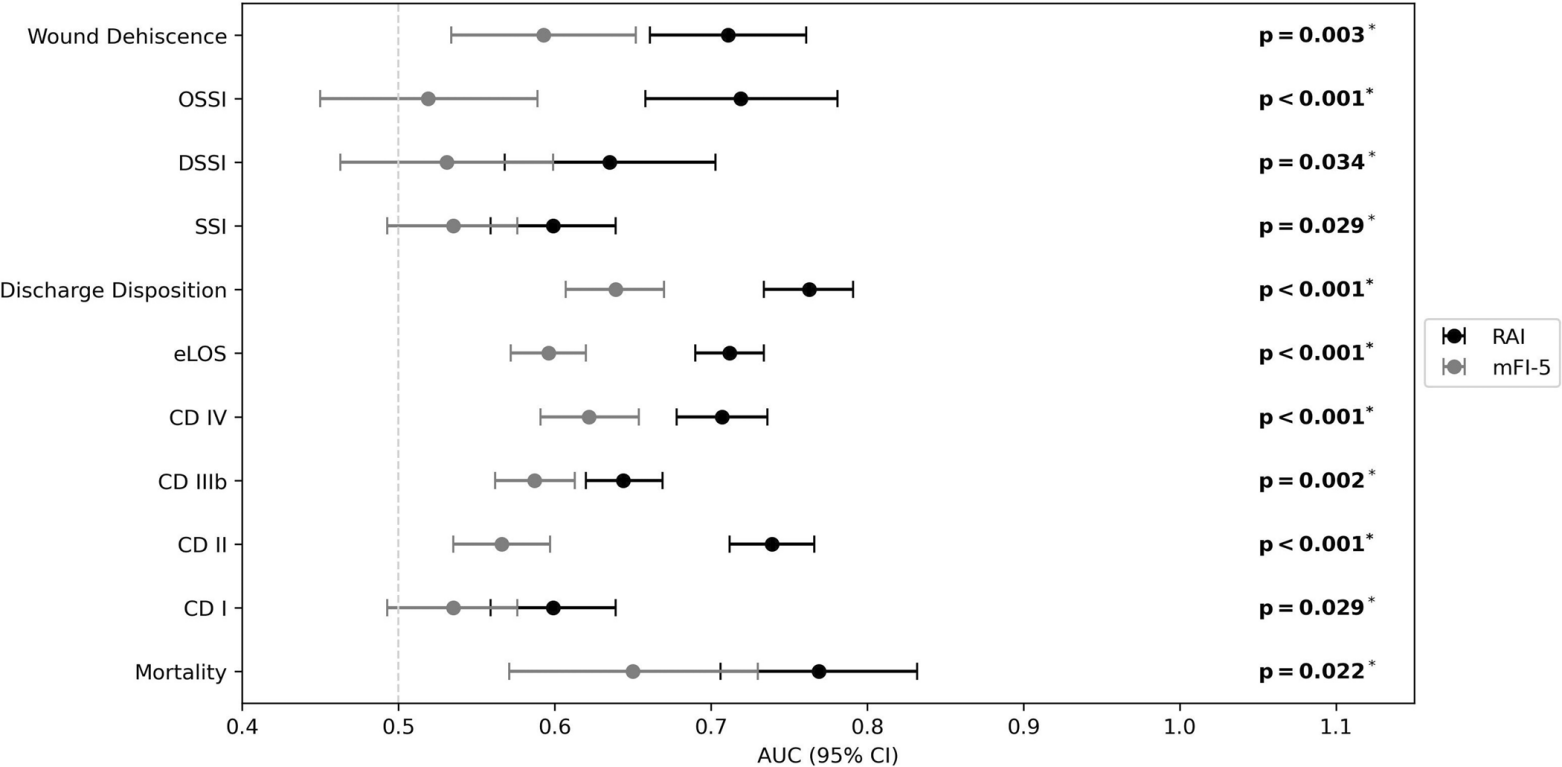
Receiver operating characteristic (ROC) forest plot with pairwise DeLong test: outcomes (*y*‐axis) with area under the curve (AUC) values (*x*‐axis) for 5‐factor Modified Frailty Index (mFI‐5) (empty squares) and Risk Analysis Index (RAI) (dark squares). Whiskers show 95% CI; *P*‐values from DeLong test; significance *P* < .05. CD, Clavien‐Dindo; DSSI, deep incisional site infection; eLOS, extended length of stay; OSSI, organ/space surgical site infection; SSI, superficial incisional site infection.

Multivariate regression controlling for age, sex, race/ethnicity, body mass index (BMI), transfer status, nonelective surgery, and operative time demonstrated that increasing frailty was independently associated with higher postoperative risk across all major outcomes (Table 3, Figure 1). Notably, RAI showed a steeper and more consistent risk gradient than mFI‐5 for mortality, with significantly elevated odds among frail (OR 8.690, 95% CI 3.896‐19.382, *P* < .001) and severely frail patients (OR 15.508, 95% CI 3.227‐74.521, *P* < .001). The mFI‐5 also predicted mortality, though with a lower gradient (severely frail: OR 10.713, 95% CI 4.232‐27.129, *P* < .001).

**Table 3:** Multivariate Analysis of Primary Outcomes Adult Thyroid and Parathyroid Surgery Patients Characterized by Frailty Screening Tool (Risk Analysis Index and Modified Frailty Index‐5, n = 30,362)

Cohort and index	Mortality (OR, 95% CI)	CD I (OR, 95% CI)	CD II (OR, 95% CI)	CD IIIb (OR, 95% CI)	CD IV (OR, 95% CI)	eLOS (OR, 95% CI)	Non‐home discharge (OR, 95% CI)
RAI pre‐frail^a^	2.781 (1.432‐5.401)*	1.158 (0.839‐1.597)	2.805 (2.109‐3.730)**	1.621 (1.318‐1.995)**	2.377 (1.824‐3.097)**	2.142 (1.760‐2.607)**	3.781 (2.904‐4.922)**
RAI frail^a^	8.690 (3.896‐19.382)**	1.173 (0.658‐2.089)	3.073 (2.049‐4.608)**	2.527 (1.776‐3.595)**	3.531 (2.386‐5.224)**	3.862 (2.929‐5.093)**	11.886 (8.417‐16.785)**
RAI severely frail^a^	15.508 (3.227‐74.521)**	5.108 (2.019‐12.921)*	7.130 (3.224‐15.769)**	5.100 (2.222‐11.707)**	4.732 (1.871‐11.965)**	9.480 (5.048‐17.082)**	15.897 (6.888‐36.689)**
mFI‐5 pre‐frail	1.244 (0.591‐2.618)	1.016 (0.719‐1.436)	1.500 (1.107‐2.031)*	1.619 (1.292‐2.208)**	1.697 (1.246‐2.311)**	1.458 (1.167‐1.822)**	1.276 (0.956‐1.703)
mFI‐5 frail^b^	1.407 (0.591‐3.349)	1.063 (0.690‐1.636)	2.023 (1.401‐2.922)**	1.893 (1.420‐2.523)**	2.224 (1.564‐3.162)**	2.043 (1.575‐2.649)**	2.288 (1.645‐3.181)**
mFI‐5 severely frail^b^	10.713 (4.232‐27.119)**	2.043 (0.949‐4.399)	3.760 (1.944‐7.272)**	3.610 (2.095‐6.223)**	6.012 (3.589‐10.071)**	7.952 (5.442‐11.617)**	9.346 (5.746‐15.200)**
Age^a^	1.06 (1.03‐1.08)**	1.010 (1.000‐1.020)*	1.04 (1.03‐1.05)**	1.01 (1.01‐1.03)**	1.03 (1.02‐1.04)**	1.03 (1.02‐1.04)**	1.05 (1.04‐1.06)**

Abbreviations: CD, Clavien‐Dindo; CD I, surgical site infection; CD II, postoperative bleeding or transfusion(s); CD IIIb, reoperation; CD IV, sepsis, septic shock, pulmonary embolism, myocardial infraction, ventilator status; eLOS, extended length of stay; mFI‐5, modified frailty index‐5; OR, odds ratio; RAI, Risk Analysis Index.

^a^ Covariates controlled for in this model include race, body mass index, primary procedure (craniectomy, craniotomy, and other), size of cranial defect (≤5 cm and >5 cm), and material (allograft, autograft, and other).

^b^ Also controlled for age in addition to the other covariates.

* *P* < .05.

** *P* < .001, statistical significance.

For eLOS and NHD, RAI demonstrated stronger associations at higher frailty levels (eLOS: OR 9.480, 95% CI 5.048‐17.082; NHD: OR 35.697, 95% CI 6.888‐186.899; both *P* < .001), compared with the corresponding mFI‐5 estimates (eLOS: OR 7.952, 95% CI 4.546‐13.946; NHD: OR 9.346, 95% CI 7.056‐45.726; *P* < .001 for both). Increasing frailty by either index was also significantly associated with higher odds of CD II to IV postoperative complications (Table 3).

### Secondary Outcomes

Among secondary complications, RAI was a stronger predictor of organ‐space infection (RAI severely frail: OR 5.487, 95% CI 1.396‐21.561, *P* < .05; mFI‐5 severely frail: OR 2.004, 95% CI 0.572‐7.025, *P* = .216), and superficial surgical site infection (RAI severely frail: OR 5.108, 95% CI 2.019‐12.921, *P* < .001; mFI‐5 severely frail: OR 2.043, 95% CI 0.949‐4.399, *P* = .068), whereas mFI‐5 more strongly predicted wound dehiscence (mFI‐5 severely frail: OR 7.498, 95% CI 2.752‐20.429, *P* < .001; RAI severely frail: OR 3.440, 95% CI 0.886‐13.350, *P* = .08) (Table 4). Associations with deep surgical site infection varied between indices and were not uniformly significant.

**Table 4:** Multivariate Analysis of Secondary Outcomes in Adult Thyroid and Parathyroid Surgery Patients Characterized by Frailty Screening Tool (Risk Analysis Index and Modified Frailty Index‐5, n = 30,362)

Cohort and index	Superficial surgical site infection (OR, 95% CI)	Deep surgical site infection (OR, 95% CI)	Organ‐space site infection (OR, 95% CI)	Wound dehiscence (OR, 95% CI)
RAI pre‐frail^a^	1.158 (0.839‐1.597)	0.966 (0.544‐1.716)	2.411 (1.347‐4.317)*	1.633 (0.969‐2.750)
RAI frail^a^	1.173 (0.658‐2.089)	1.673 (0.753‐3.724)	2.677 (1.119‐5.979)*	2.044 (0.990‐4.220)
RAI severely frail^a^	5.108 (2.019‐12.921)**	3.246 (0.687‐15.343)	5.487 (1.396‐21.561)*	3.440 (0.886‐13.350)
mFI‐5 pre‐frail^b^	1.016 (0.719‐1.436)	1.191 (0.661‐2.145)	0.908 (0.509‐1.620)	2.435 (1.357‐ 4.368)*
mFI‐5 frail^b^	1.063 (0.690‐1.636)	0.820 (0.365‐1.843)	0.943 (0.44‐2.00)	2.112 (1.001‐ 4.457)*
mFI‐5 severely frail^b^	2.043 (0.949‐4.399)	2.153 (0.611‐7.582)	2.004 (0.572‐7.025)	7.498 (2.752‐20.429)**
Age^a^	1.01 (1.00‐1.02)*	1.01 (1.00‐1.01)	1.03 (1.01‐1.05)**	1.02 (1.01‐1.04)**

Abbreviations: mFI‐5, Modified Frailty Index‐5; OR, odds ratio; RAI, Risk Analysis Index.

^a^ Covariates controlled for in this model include race, body mass index, primary procedure (craniectomy, craniotomy, and other), size of cranial defect (≤5 cm and >5 cm), and material (allograft, autograft, and other).

^b^ Also controlled for age in addition to the other covariates.

* *P* < .05.

** *P* < .001, statistical significance.

### ROC Curve Analysis and *C*‐Statistics

ROC analysis demonstrated that RAI consistently outperformed mFI‐5 in discriminating postoperative morbidity across multiple outcomes (Table 5, Figure 1). RAI showed significantly higher *C*‐statistics for eLOS (0.712 vs 0.596, *P* < .001), NHD (0.763 vs 0.639, *P* < .001), CD II complications (0.739 vs 0.566, *P* < .001), CD IIIb (0.644 vs 0.587, *P* = .002), CD IV (0.707 vs 0.622, *P* < .001), and organ‐space infection (0.719 vs 0.519, *P* < .001). For mortality, both indices demonstrated significant discriminatory ability, with comparable performance between RAI (0.769, 95% CI 0.706‐0.832) and mFI‐5 (0.650, 95% CI 0.571‐0.730) (*P* = .022).

**Table 5:** Receiver Operating Characteristic (ROC) Analysis *C*‐Statistics of Primary Outcomes in Adult Thyroid/Parathyroid Surgery

Outcome variables	RAI AUC (95% CI)	mFI‐5 AUC (95% CI)	Age AUC (95% CI)
Mortality	0.769 (0.706‐0.832)**	0.650 (0.571‐0.730)**	0.701 (0.634‐0.768)**
Extended length of stay	0.712 (0.690‐0.734)**	0.596 (0.572‐0.620)**	0.612 (0.590‐0.635)**
Discharge disposition	0.763 (0.734‐0.791)**	0.639 (0.607‐0.670)**	0.694 (0.665‐0.723)**
Surgical site infection	0.599 (0.559‐0.639)**	0.535 (0.493‐0.576)	0.540 (0.502‐0.578)*
Organ‐space infection	0.719 (0.658‐0.781)**	0.519 (0.450‐0.589)	0.635 (0.576‐0.694)**
Deep surgical site infection	0.635 (0.568‐0.703)**	0.531 (0.463‐0.599)	0.577 (0.513‐0.642)*
Wound dehiscence	0.711 (0.661‐0.761)**	0.593 (0.534‐0.652)*	0.608 (0.562‐0.653)*
CD I	0.599 (0.599‐0.639)**	0.535 (0.493‐0.576)	0.540 (0.502‐0.578)*
CD II	0.739 (0.712‐0.766)**	0.566 (0.535‐0.597)**	0.661 (0.634‐0.687)**
CD IIIB	0.644 (0.620‐0.669)**	0.587 (0.562‐0.613)**	0.584 (0.560‐0.607)**
CD IV	0.707 (0.678‐0.736)**	0.622 (0.591‐0.654)**	0.626 (0.597‐0.656)**

Abbreviations: AUC, area under the curve; CD, Clavien‐Dindo, CD II, postoperative bleeding or transfusion(s); CD IIIB, reoperation; CD IV, sepsis, septic shock, pulmonary embolism, myocardial infraction, ventilator status; eLOS, extended length of stay; mFI‐5, Modified Frailty Index‐5; RAI‐A, Risk Analysis Index.

* *P* < .05.

** *P* < .001, statistically significant difference from the threshold of no‐difference (.5).

## Discussion

This is the first study to evaluate the RAI in thyroid and parathyroid surgery and the first to compare RAI and mFI‐5 in any low‐risk otolaryngology population. Both indices demonstrated significant predictive capacity for adverse events; however, the RAI both produced higher ORs and showed steeper and more consistent risk gradients across all primary, and most secondary outcomes, including mortality, eLOS, and NHD. The RAI also demonstrated superior discrimination for every morbidity outcome on ROC analysis, underscoring a stronger ability to correctly classify high‐risk patients despite a low‐acuity procedural cohort. Together, these findings suggest that although both instruments capture meaningful frailty‐related risk, the RAI offers greater clinical and practical utility in preoperative risk stratification for thyroid/parathyroid surgery. Evaluating frailty in this context is not redundant; this study acts as a litmus test of whether these instruments can meaningfully stratify risk when baseline complication rates are low and physiological stressors are minimal.

Frailty has been associated with adverse postoperative outcomes across otolaryngology but also within specific surgeries such as head and neck cancer surgery, thyroid surgery for multinodular goiters, thyroidectomies, and parathyroidectomies.^13^, ^14^, ^15^ The relative prevalence of studies on the association between frailty and postoperative outcomes across a wide range of surgical specialties highlights the significance of physiological age in surgical outcomes rather than chronological age alone. In otolaryngology, numerous frailty indices have been studied, including mFI‐5 (most prevalent), RAI, mFI‐11, Johns Hopkins Adjusted Clinical Groups (ACG) indicator, and Clinical Frailty Scale (CFS).^14^, ^15^, ^16^, ^17^, ^18^ Despite the availability of numerous frailty assessment tools, there is a lack of consensus on which of them provides the most accurate risk stratification preoperatively based on their association with postoperative outcomes. Additionally, despite the presence of existing studies on frailty in otolaryngology surgeries, there is a paucity of studies that compare the use of multiple frailty tools in their association with postoperative outcomes.

Several important conclusions can be drawn from this study's results, demonstrating the superiority of the RAI to the mFI‐5 in multivariable and ROC models. First, our findings reinforce that frailty, not chronological age, is a central determinant of postoperative vulnerability in endocrine surgery. Prior literature has demonstrated that diminished physiologic reserve compromises wound healing, immune response, and metabolic resilience, thereby increasing susceptibility to adverse events such as infection and delayed recovery.^17^, ^19^ Consistent with this framework, both frailty indices were independently associated with multiple postoperative outcomes in our cohort, underscoring the relevance of frailty assessment even in low‐acuity thyroid and parathyroid procedures.

Second, the superior performance of the RAI across both multivariable and ROC models likely reflects fundamental differences in index design. The mFI‐5 captures only five binary comorbidities, each weighted equally, which limits its ability to detect gradations in vulnerability that arise from functional or cognitive decline. In contrast, the RAI integrates multidomain factors, including functional dependence, dyspnea, nutritional status, residential status, and comorbidities, each with independent weighting.^2^ This structure allows the RAI to quantify frailty with greater physiologic fidelity, which may explain its steeper risk gradient and significantly better discrimination for eLOS, NHD, organ‐space infection, and CD II to IV complications. Similar findings have been reported across neurosurgical and other surgical populations, where the RAI consistently outperforms comorbidity‐based indices due to its ability to capture the multidimensional construct of frailty.^2^, ^20^, ^21^ In addition, while mFI‐5 scales each variable equally, RAI scales each parameter independently, which allows for a better accounting of the physiological reserve of the patient.^2^, ^8^, ^22^, ^23^, ^24^

Finally, although prior endocrine surgery studies, such as Taylor et al and Finnerty et al, have validated the mFI‐5 as a predictor of postoperative complications, they did not compare it against multidomain frailty instruments and relied on smaller or older cohorts.^23^, ^24^ This study updates and expands this literature by using a large, contemporary data set to directly compare mFI‐5 with the RAI, demonstrating clear and clinically meaningful advantages of the RAI across both effect‐size estimates and discrimination metrics.^25^, ^26^ These findings align with recent evidence from Evans et al showing strong predictive performance of the RAI in otolaryngology more broadly.^13^ Collectively, this study provides the first head‐to‐head comparison of RAI versus mFI‐5 in thyroid and parathyroid surgery and establishes a foundation for incorporating multidimensional frailty assessment into preoperative risk stratification for low‐risk otolaryngology populations.

As the surgical population ages, the prevalence of frailty will continue to rise, and preoperative identification of vulnerable patients will remain essential.^27^ Although thyroid and parathyroid surgeries are generally low‐risk, anticipating which patients may require postoperative observation, NHD planning, or extended recovery is clinically meaningful.^26^, ^27^, ^28^ Frailty assessment has traditionally been used in high‐risk or non‐elective settings, but emerging evidence, including the present study, demonstrates its value in elective procedures where subtle gradients in physiologic reserve shape outcomes.^2^, ^29^, ^30^, ^31^, ^32^ Evidently, frailty should not be used to deny surgery in scenarios such as advanced head and neck cancer, where operative management is unavoidable.^31^, ^33^ However, in select endocrine operations or low‐risk thyroid cancer, frailty assessment can meaningfully influence decisions regarding operative timing, surgical aggressiveness, prehabilitation, and postoperative support planning. Importantly, despite containing more weighted variables, the RAI is not burdensome to implement (completion typically requires under 30 seconds in clinical practice), making its modest but clinically meaningful improvements in predictive accuracy worthwhile in low‐risk elective surgery.^34^, ^35^

Future research should prospectively validate these findings across diverse otolaryngology populations and examine the integration of frailty screening into routine preoperative workflows. Investigating the effect of targeted prehabilitation interventions, such as nutrition optimization and exercise therapy, on frail patients undergoing endocrine surgery may further refine perioperative care. Comparative evaluation of RAI and mFI‐5 alongside other geriatric frailty tools, such as the CFS or Fried phenotype, could further clarify optimal screening strategies.

This study has a few limitations. Thyroidectomy and parathyroidectomy were analyzed together because more than 90% of cases had overlapping CPT coding, limiting the feasibility of fully separate models, and NSQIP does not reliably distinguish primary, secondary, and tertiary hyperparathyroidism. Patients >90 years were excluded due to NSQIP top‐coding, and 0.6% with missing functional status were removed. Frailty was calculated using the RAI‐A rather than the clinical RAI‐C, and some overlap between frailty components may contribute to minor multicollinearity. Although the RAI incorporates variables that are routinely collected in standard preoperative assessment (eg, dyspnea, ADLs, comorbidities, and residence status), certain elements, such as unintentional weight loss or poor appetite, may be documented inconsistently in retrospective data sets. As with all retrospective database studies, unmeasured confounding and lack of causal inference remain unavoidable.

## Conclusion

In this large, retrospective cohort study, frailty, whether measured by the RAI or mFI‐5, was a significant and independent predictor of postoperative morbidity following thyroid and parathyroid surgery. Although both indices captured meaningful physiologic vulnerability, the RAI demonstrated consistently stronger effect‐size gradients and superior discriminatory performance for multiple clinically relevant outcomes, including eLOS, NHD, and organ‐space infection. These findings reinforce that physiological reserve, not chronological age, is central to postoperative risk, even in low‐acuity elective procedures. Importantly, the ability of the RAI to stratify risk in a setting characterized by minimal operative stress and low baseline complication rates suggests that its utility extends beyond high‐risk or emergent surgery and may offer broader relevance across elective otolaryngologic and surgical care. By demonstrating that a multidomain frailty instrument outperforms a comorbidity‐based index in this context, this study provides new evidence supporting the routine incorporation of frailty assessment into preoperative decision‐making for endocrine neck surgery. Prospective validation in diverse populations and evaluation of prehabilitation or targeted optimization strategies will further clarify how frailty‐informed risk stratification can improve perioperative planning, shared decision‐making, and postoperative recovery. Ultimately, these findings underscore RAI frailty assessment as a uniquely important tool for advancing personalized, evidence‐based surgical care in thyroid and parathyroid surgery.

## Author Contributions

**Akshay Warrier**, Project ideation, data collection, data analysis, writing; **Sruthi Ranganathan**, Data analysis, writing; **Deondra Montgomery**, Writing; **Jonathan Tawil**, Writing; **Ari Istanboulli**, Writing; **Christian Bowers**, Project ideation, editing; **Richard K. Gurgel**, Project ideation, editing; **Hilary McCrary**, Project ideation, editing.

## Disclosures

### Competing interests

The authors declare no conflicts of interest.

### Funding source

None.

Abstract Presented at the 2025 COSM Triological Society Annual Meeting; New Orleans, Louisiana; May 15, 2025.

## Supporting information

Supplemental Table S1: Current Procedural Terminology (CPT) codes for patient selection. CPT codes identifying thyroidectomy and parathyroidectomy cases with procedure descriptions.

Supplemental Table S2: Clavien‐Dindo complication classifications. Classification of postoperative complications from grade I (minor) to grade V (death), adapted from Clavien et al.
