# Supplementary material for: Risk Analysis Index Outperforms the Modified Frailty Index in Predicting Outcomes in Thyroidectomy and Parathyroidectomy
Source: Otolaryngol Head Neck Surg. 2026 Jan 19;174(3):705–14. doi: 10.1002/ohn.70125 (PMC12948393; doi:10.1002/ohn.70125)
Supplement: Supplementary file 1 — Supplemental Table S1: Current Procedural Terminology (CPT) codes for patient selection. CPT codes identifying thyroidectomy and parathyroidectomy cases with procedure descriptions. [file OHN-174-705-s001.docx]

**Supplemental Table 1:** CPT Codes for Patient Selection

| **CPT Code** | **Procedure Description** |
| --- | --- |
| **60200** | Excision of cyst or adenoma of thyroid, or transection of isthmus |
| **60210** | Partial thyroid lobectomy, unilateral; with or without isthmusectomy |
| **60212** | Partial thyroid lobectomy, unilateral; with contralateral subtotal lobectomy, including isthmusectomy |
| **60220** | Total thyroid lobectomy, unilateral; with or without isthmusectomy |
| **60225** | Total thyroid lobectomy, unilateral; with contralateral subtotal lobectomy, including isthmusectomy |
| **60240** | Thyroidectomy, total or complete |
| **60252** | Thyroidectomy, total or subtotal for malignancy; with limited neck dissection |
| **60260** | Removal of all remaining thyroid tissue following previous removal of a portion of thyroid |
| **60500** | Parathyroidectomy or exploration of parathyroid(s) |
| **60502** | Parathyroidectomy or exploration of parathyroid(s); re-exploration |
| **60512** | Parathyroid autotransplantation (List separately in addition to code for primary procedure) |
