# Supplementary material for: Risk Analysis Index Outperforms the Modified Frailty Index in Predicting Outcomes in Thyroidectomy and Parathyroidectomy
Source: Otolaryngol Head Neck Surg. 2026 Jan 19;174(3):705–14. doi: 10.1002/ohn.70125 (PMC12948393; doi:10.1002/ohn.70125)
Supplement: Supplementary file 2 — Supplemental Table S2: Clavien‐Dindo complication classifications. Classification of postoperative complications from grade I (minor) to grade V (death), adapted from Clavien et al. [file OHN-174-705-s002.docx]

**Supplemental Table 2**: Clavien-Dindo Complication Classifications

| **Grade** | **Definition** |
| --- | --- |
| Grade I | Deviation from the normal postoperative course without pharmacological treatment or surgical, endoscopic, and radiological interventions. Included interventions: drugs such as antiemetics, antipyretics, analgesics, diuretics, and electrolytes, as well as physiotherapy. Wound infections opened at the bedside also included. |
| Grade II | Requiring pharmacological treatment with drugs other than those allowed for Grade I complications. Blood transfusions and total parenteral nutrition are also included. |
| Grade III | Requiring surgical, endoscopic, or radiological intervention. |
| *Grade III-a* | Intervention not under general anesthesia. |
| *Grade III-b* | Intervention under general anesthesia. |
| Grade IV | Life-threatening complication (including CNS complications) requiring ICU management. |
| *Grade IV-a* | Single organ dysfunction (including dialysis). |
| *Grade IV-b* | Multiorgan dysfunction. |
| Grade V | Death of a patient. |
